# Transient Femoral Nerve Palsy Complicating “Blind” Transversus Abdominis Plane Block

**DOI:** 10.1155/2013/874215

**Published:** 2013-09-04

**Authors:** Dimitrios K. Manatakis, Nikolaos Stamos, Christos Agalianos, Michail Athanasios Karvelis, Michael Gkiaourakis, Demetrios Davides

**Affiliations:** ^1^1st Surgical Department, Athens Naval and Veterans Hospital, 70 Dinokratous Street, 11521 Athens, Greece; ^2^Department of Anesthesiology, Athens Naval and Veterans Hospital, 70 Dinokratous Street, 11521 Athens, Greece

## Abstract

We present two cases of patients who reported quadriceps femoris weakness and hypoesthesia over the anterior thigh after an inguinal hernia repair under transversus abdominis plane (TAP) block. Transient femoral nerve palsy is the result of local anesthetic incorrectly injected between transversus abdominis muscle and transversalis fascia and pooling around the femoral nerve. Although it is a minor and self-limiting complication, it requires overnight hospital stay and observation of the patients. Performing the block under ultrasound guidance and injecting the least volume of local anesthetic required are ways of minimizing its incidence.

## 1. Introduction

Transient femoral nerve palsy (TFNP) occurs in 5–8% of ilioinguinal/iliohypogastric nerve (IIN/IHN) blocks [1, 2]. To the best of our knowledge it has been reported only once following a transversus abdominis plane (TAP) block [3].

We present our experience with 2 cases of TFNP complicating “blind” TAP blocks for inguinal herniorrhaphy, investigate its mechanism, and discuss possible ways of prevention. 

## 2. Case 1

An otherwise healthy, 26-year-old, nonobese, male patient was scheduled to undergo right inguinal hernia plug-and-patch repair as a day case, under TAP block anesthesia and conscious sedation. Twenty mL of 0,5% ropivacaine were administered by an experienced anesthesiologist, using the landmark-based “two-pop” technique as described by McDonnell et al. [4]. Surgery was uneventful, with a duration of 45 minutes.

At the postoperative ward round, the patient was disturbed and reported inability to extend the ipsilateral knee joint. Clinical examination revealed quadriceps femoris paresis, hypoesthesia over the anterior aspect of the thigh, and absent patellar reflex. The patient and his family were reassured, and the self-limiting nature of the complication was explained. He was admitted overnight for observation. On the following morning, symptoms had completely remitted, and he was discharged.

## 3. Case 2

A 62-year-old, nonobese, male patient, with an unremarkable past medical history, was scheduled to undergo left inguinal hernia plug-and-patch repair as a day case, under TAP block anesthesia and conscious sedation. Twenty mL of 0,5% ropivacaine were injected into the TAP by the same anesthesiologist. Surgery was uneventful, with a duration of 50 minutes.

Two hours postoperatively he suffered from a minor orthopedic injury (ankle sprain) on his attempt to stand up from bed. On clinical examination he was found to have quadriceps femoris muscle weakness (grade 1/5 according to Louisiana State University Health Services Center grading system) and hypoesthesia of the anterior thigh. Symptoms lasted for 8 hours. The patient was admitted overnight, recovered fully, and was discharged on the following morning.

## 4. Discussion

Transversus abdominis plane (TAP) is the anatomic space between the transversus abdominis and internal oblique muscles. Its clinical significance lies in the fact that it is traversed by the nerves that provide sensory supply to the anterolateral abdominal wall (T7-T11 intercostal, subcostal, iliohypogastric, and ilioinguinal nerves) [5, 6].

TAP is approached traditionally through the lumbar triangle of Petit by a “blind” landmark-based technique or in the midaxillary line under ultrasound guidance [4, 7, 8]. TAP block provides regional lower abdominal anesthesia and is mainly used as a component of multimodal postoperative analgesia regimens [9].

It is generally considered a safe procedure with only few cases of complications found in the literature [10, 11]. A thorough search of the English-speaking literature revealed only one case of TFNP following a TAP block. In a letter to the editor, Dr. Walker briefly described a case of a patient fracturing their ankle, when trying to mobilize from bed [3]. 

In their cadaveric study Rosario et al. found that at the level of the anterior superior iliac spine the femoral nerve lies in the groove formed by the psoas major and the iliacus muscles, covered by the iliacus fascia. The authors demonstrated that the iliacus fascia is the posterolateral continuation of the transversalis fascia, meaning that the femoral nerve lies in the same tissue plane as the space deep to the transversus abdominis muscle [12]. Therefore advancement of the needle and injection of local anesthetic into the wrong tissue plane, that is, between the transversus abdominis muscle and the transversalis fascia (instead of the plane between the internal oblique and transversus abdominis muscles), lead to the injectate tracking along the transversalis fascia and eventually accumulating around the femoral nerve.

The ensuing femoral nerve palsy results in quadriceps femoris paresis or weakness and hypoaesthesia over the anteromedial thigh that usually last for 6 to 8 hours (up to 36 hours in one case report [13]) and resolve spontaneously without permanent neurological deficit. 

Another possible mechanism proposed by Rosario et al. is the direct injection of local anaesthetic around the femoral nerve, if the injection is performed 3-4 cm more medially than that recommended for IIN/IHN blocks [12]. Walker warns that injection point for blind TAP block is even more posterior and likely closer to the femoral nerve trunk [3]. Interestingly, the distance between needle point and femoral nerve was found longer in females than in males, making TFNP perhaps less likely to occur in female patients [12].

Kulacoglu et al. drew the same conclusions in their cadaveric study [14]. They further suggest that a step-by-step infiltration technique under direct surgical vision is safer than blind blocks. Ghani et al. however failed to show a difference in TFNP incidence between anaesthetists who performed blind blocks preoperatively and surgeons who performed blocks under direct vision intraoperatively [1]. McDermott et al. used ultrasound to assess needle position and administration of local anaesthetic. They found that the local anaesthetic was correctly delivered into the TAP only in 23,6% of patients, while in 30% it was injected deeper than required [15]. 

A second aspect of the mechanism of TFNP is the choice and dosage of local anaesthetic. Rosario et al., using methylene blue as injectate, demonstrated that as little as 1 mL of dye injected in the wrong tissue plane was sufficient to pool around the femoral nerve, while Kulacoglu et al. used a volume of 10 mL to better match a clinical scenario [12, 14]. Given the different tissue conditions in vivo, safe conclusions can not be drawn from cadaveric studies, as to the volume and concentration of local anaesthetic required to produce TFNP. 

Epperson and Reese reported a case of TFNP, following a field block with 20 mL of 0,5% bupivacaine and 1 : 200000 epinephrine after inguinal herniorrhaphy, which led only to femoral sensory neuropraxia with preservation of normal motor function [16]. Similarly Wulf et al. reported consistent sensory blockade with all examined dosing schemes of ropivacaine but motor blockade only at higher dosages, implying use of the least amount and lowest concentration of local anaesthetic possible, to minimise TFNP incidence [17]. To date, there is insufficient evidence to support any particular local anaesthetic, and the ideal combination and dosage have yet to be determined [9, 18].

Essentially the majority of TAP block complications and failures is the result of wrong needle placement and local anaesthetic injection either too deep or too superficial [16, 19]. Performing the block under real-time ultrasound guidance is a valid option. Advancement of needle and injection of local anaesthetic are more accurate than those by “blind” techniques [6, 9, 20]. Ultrasound-guided infiltration has also been reported to achieve faster absorption and higher plasma concentrations of local anaesthetic, suggesting that smaller volumes may be required, for equal anaesthetic effect [21–23].

Hebbard and Shibata approach the TAP by placing the ultrasound probe transversely across the midaxillary line, where the layers of the abdominal wall can be more easily visualised [8, 24]. With this technique the needle is introduced more anteriorly than the triangle of Petit and further from the femoral nerve. Moreover, using a blunt-tipped needle and advancing it obliquely instead of perpendicularly increase the resistance of each aponeurotic layer, making the “pop” sensation of fascial piercing more discernible [25]. 

## 5. Conclusion

TFNP after TAP block is the result of local anaesthetic incorrectly injected between the transversus abdominis muscle and the transversalis fascia and accumulating around the femoral nerve. While it is not a major cause of postoperative morbidity, it may cause patient discomfort and anxiety as well as unexpected injuries due to falls. It is a self-limiting complication, but patients require overnight admittance for observation, thus increasing length of stay and hospital costs. Performing the TAP block under ultrasound guidance and injecting the least volume of local anaesthetic required are effective ways to reduce its occurrence.
